# Cannabis use for exercise recovery in trained individuals: a survey study

**DOI:** 10.1186/s42238-023-00198-5

**Published:** 2023-08-05

**Authors:** Anthony G. Pinzone, Emily K. Erb, Stacie M. Humm, Sarah G. Kearney, J. Derek Kingsley

**Affiliations:** 1 Exercise Science and Exercise Physiology, Kent State University, Kent, OH 44242 USA; 2 Grand Valley State University, Allendale, MI 49401 USA

**Keywords:** Delta-9-tetrahydrocannabinol, Cannabidiol, Aerobic exercise, Recovery

## Abstract

**Background:**

Cannabis use, be it either cannabidiol (CBD) use and/or delta-9-tetrahydrocannabinol (THC) use, shows promise to enhance exercise recovery. The present study aimed to determine if individuals are using CBD and/or THC as a means of recovery from aerobic and/or resistance exercise, as well as additional modalities that might be used to aid in recovery.

**Methods:**

Following consent, 111 participants (Mean ± SD: Age: 31 ± 13 years) completed an anonymous survey. All participants were regularly using cannabis (CBD and/or THC) as well as were currently exercising. Questions pertained to level of cannabis use, methods used for consumption of cannabis, exercise habits, exercise recovery strategies, and demographics.

**Results:**

Eighty-five percent of participants reported participating in aerobic training. In addition, 85% of participants also reported regular participation in resistance exercise. Seventy-two percent of participants participated in both aerobic and resistance exercise. Ninety-three percent of participants felt that CBD use assisted them with recovery from exercise, while 87% of participants felt the same regarding THC use.

**Conclusions:**

Individuals who habitually use cannabis, CBD or THC, and regularly engage in exercise do feel that cannabis assists them with exercise recovery. More data are necessary to understand the role of cannabis in exercise recovery as well as perceived ergogenic benefits of cannabis by individuals who both regularly participate in exercise and habitually use cannabis.

**Supplementary Information:**

The online version contains supplementary material available at 10.1186/s42238-023-00198-5.

## Background

Over the past decade, cannabis use has become more widespread in the United States, both medically and recreationally (YorkWilliams et al. 2019). As of 2021, 52.5 million individuals in the US reported use of cannabis in the past year, accounting for 18.7% of the country’s population (Key substance use and mental health indicators in the United States: Results from the 2021 national survey on drug use and health 2021). Cannabis plants are composed of a variety of cannabinoid compounds, most notably cannabidiol (CBD) and delta-9-tetrahydrocannabidiol (THC), both of which bind endocannabinoid receptors, cannabinoid type 1 (CB1) and cannabinoid type 2 (CB2) (Pagotto et al. 2006). While CBD has a low-binding affinity for CB1 and CB2 receptors, both receptors bind THC (Camilleri 2018). Contrary to THC, CBD does not induce psychotropic effects (Baranowska-Kuczko et al. 2020). It has, however, been demonstrated to acutely stimulate parasympathetic nervous system activation resulting in reduced heart rate, reduced systolic blood pressure, and increased vasodilation (Baranowska-Kuczko et al. 2020). Conversely, THC has been demonstrated to impair cognitive function and up-regulate sympathetic nervous system activity, leading to acute increases in heart rate, systolic blood pressure, and vasoconstriction (Zhornitsky et al. 2021; Kaufmann et al. 2010).

CBD and THC have the potential to enhance recovery from aerobic and resistance exercise due to analgesic, anti-inflammatory effects as well as the ability to enhance sleep quality (YorkWilliams et al. 2019). Both compounds have also assisted in acutely reducing subjective feelings of pain intensity in chronic-pain patients, while acute CBD intake has been demonstrated to attenuate muscle damage following resistance exercise in resistance-trained men and women (Almog et al. 2020; Isenmann et al. 2021). Survey data in both recreationally active and athletic populations has demonstrated that individuals use cannabis to assist with exercise recovery, pain relief resulting from muscle-soreness, to reduce inflammation, and enhance sleep (YorkWilliams et al. 2019). Further survey data also suggests that cannabis-using athletes feel that THC and CBD use can promote feelings of relaxation and enhanced well-being with minimal perceived adverse effects (Zeiger et al. 2019). It is key to note, however, that some CBD preparations contain trace amounts of THC, potentially resulting in a positive test for drug use in athletic populations (Lachenmeier and Diel 2019) or those required to take a drug test as part of their employment. A review by Docter et al. (2020) reported that athletes using cannabis felt that it improved sleep and reduced anxiety and also reiterated the dearth of knowledge on how cannabis affects recovery (Docter et al. 2020). Additionally, both CBD and THC use have been demonstrated to acutely down-regulate release of pro-inflammatory cytokines such as tumor necrosis factor α (TNF- α) and interleukin (IL)-12 (Ajrawat et al. 2022). Furthermore, habitual cannabis users have displayed lower circulating levels of C-reactive protein (CRP), an indicator of global inflammation, when compared to non-users (Alshaarawy and Anthony 2015). Sahinovic et al. (2022) reported possibly decreased cytokines TNF- α and IL-6 when CBD was taken prior to submaximal aerobic exercise (Sahinovic et al. 2022). Lastly, chronic cannabis use has been observed to assist individuals with insomnia, attenuating symptoms and assisting in falling and remaining asleep with greater benefits observed with THC when compared to CBD (Kuhathasan et al. 2021). Therefore, multiple acute and chronic effects of using CBD or THC make these substances potentially promising interventions for enhancing recovery from aerobic and resistance exercise.

Other than cannabis, ancillary recovery modalities such as stretching, heat therapy (hot tub and sauna), foam rolling, cupping, electrical stimulation, compression garments, cryotherapy, contrast water bath, cold-water immersion and other supplements focused on recovery have all been purported to enhance exercise recovery as well that are highlighted in a review by Dupuy et al. (2018). Data from this review suggests that many of these recovery modalities are used to attenuate muscle damage, reduce inflammation and feelings of perceived muscle soreness, and diminish feelings of fatigue associated following performance of exercise with varying levels of efficacy based on the modality implemented (Dupuy et al. 2018). Consequently, habitual cannabis users who also exercise may also be using cannabis to assist with exercise recovery given the ability of cannabis to reduce inflammation and assist with acute and chronic pain, similar to these ancillary recovery modalities (Almog et al. 2020; Ajrawat et al. 2022).

To date, few investigations have directly evaluated changes in muscle soreness or recovery with ingestion of cannabis surrounding aerobic or resistance exercise (Isenmann et al. 2021). Currently, there is minimal evidence regarding perceived ability of cannabis, CBD or THC, to enhance exercise recovery among individuals who habitually use cannabis and exercise regularly. Therefore, the purpose of the present investigation was to determine if individuals are utilizing cannabis to recover from aerobic or resistance exercise. We hypothesized that individuals who use cannabis, CBD or THC, and exercise regularly, would report perceived benefits of cannabis for recovery from both aerobic and resistance exercise. We also hypothesized that motives for cannabis use would include pain management, reductions in anxiety, and to improve sleep.

## Methods

A voluntary, anonymous online questionnaire entitled, ‘Cannabis and Exercise’ was advertised on social media (Instagram, Twitter, Facebook), through word of mouth, and posted flyers. All participant recruitment material contained a QR code linked to the survey. The data were collected from June 2022-November 2022. Inclusion criteria included being at least 18 years of age, currently consuming cannabis, and currently engaged in regular exercise. Once individuals read the informed consent they selected ‘yes’, or ‘no’. If the individuals selected ‘yes’ they were given access to the questionnaire, allowing participants to answer questions pertaining to cannabis use (CBD and THC), exercise habits (aerobic and resistance exercise), views on recovery methods, and demographics. If the individuals selected ‘no’ then the questionnaire ended. Participants were able to skip questions as they saw fit, and all data were anonymous. This study was approved by the Kent State University Institutional Review Board.

### Questionnaire

The questionnaire utilized multiple question formats including multiple choice, Likert scale, and check all that apply (cannabis habits, exercise habits, exercise habits in relation to cannabis use, CBD use, THC use, methods of recovery, demographics). Once consent was provided by the participant, the first part of the questionnaire was completed regarding cannabis use; one set of questions pertaining to the use of CBD, and another set pertaining to the use of THC. Each set of questions had five subsets; the duration participants have been using, number of days per week, number of times per day, amount of money spent on these products, and preferred method of consumption.

The second set of questions pertained to current exercise habits and included nine subsets; currently physically active/exercise, duration participant has been exercising, exercise with a team, minutes of exercise per week, preferred modalities of exercises, engagement in aerobic exercise, engagement in resistance exercise, the use of supplements to enhance performance, and additional recovery methods that do not include cannabis. Participants were also asked if they used cannabis as a recovery modality and were also asked if they felt that cannabis aided in recovery. There were also a set of questions related to intensity of the workouts, the number of days spent exercising, and how many minutes per week for both aerobic exercise and resistance exercise.

### Statistics

Differences between men, women, and non-binary individuals were assessed with a Kruskal–Wallis nonparametric test. Chi-square (X^2^) was used to evaluate differences between CBD users and THC users on exercise habits. In addition, Pearson’s correlation coefficients were used to assess the relationship between motivations for cannabis use and recovery. All statistical analyses were completed using IBM SPSS (Version 25, Armonk, NY, USA). All data are presented as mean ± standard deviation. Statistical significance was set a priori at p ≤ 0.05 for all comparisons.

## Results

### Participants

One hundred eleven participants completed the survey. All participants that completed the survey stated they exercised and used cannabis regularly. Fifty-nine percent of participants were women, 39% were men and 2% were non-binary. Demographic description of these groups can be noted in Table 1. Body mass index was calculated as kg/m^2^. In general, ages of participants ranged from 18–68 years, with a mean age of 31 ± 13 years. Eighty-five percent identified as Caucasian, 4% as African American, 3% as Asian, and 8% as other. Academic backgrounds included 26% with high school diploma/GED, 4% technical program certification, 6% associate degree, 34% undergraduate degree, 18% master’s degree, and 12% of participants had a doctoral degree. The average yearly income amongst participants was $25 k to $99 k.

**Table 1:** Participant characteristics (*N* = 111)

Variables	Men (*n* = 44)	Women (*n* = 65)	Non-binary (*n* = 2)
Age (yrs)	30 ± 12	33 ± 14	30 ± 11
Height (m)	1.79 ± 6.9	1.65 ± 8.0	1.64 ± 5.4
Weight (kg)	80.5 ± 10	69.5 ± 13.6	53.1 ± 5
Body mass index (kg/m^2^)	25.1 ± 2.1	25.5 ± 1.3	19.8 ± 2.7
Annual Income ($)	25-99 k	25-99 k	25-99 k

### Exercise habits

Eighty-five percent of participants reported they regularly performed aerobic exercise and 85% reported they resistance trained, with 72% participating in both modalities. Data regarding sex specific differences in exercise habits are presented in Table 2. Generally, participants that aerobically trained (*n* = 72) performed moderate-vigorous aerobic exercise, three-four days a week for 31–150 min. Participants (*n* = 72) that resistance trained reported doing so three days a week for 31–150 min/wk at a vigorous intensity. When comparing CBD users to THC users it was noted that men CBD users preferred a higher intensity for their aerobic (X^2^(4, *N* = 72) = 21.7, *p* = 0.001) and resistance exercise (X^2^(5, *N* = 72) = 10.1, *p* = 0.017), and women CBD users preferred a higher intensity resistance exercise (X^2^(5, *N* = 72) = 29.8, *p* = 0.001). There were no differences in those that identified as non-binary. Exercise modalities mentioned by the participants are included in Table 3. The choices of exercise based on rank are similar between those who use CBD and those who use THC.

**Table 2:** Exercise habits in those that use CBD and THC (*N* = 111)

**CBD Users (*n* = 20)**			
Aerobic Exercise			
	Frequency (d/wk)	Intensity	Time (min/wk)
Men (*n* = 9)	4 ± 2	Very Hard	31–150
Women (*n* = 11)	3 ± 2	Vigorous	31–150
Non-binary (*n* = 1)	5 ± 2	Vigorous	31–150
Resistance Exercise			
Men (*n* = 9)	4 ± 2	Very Hard	> 150
Women (*n* = 11)	3 ± 1	Very Hard	31–150
Non-binary (*n* = 1)	3 ± 1	Vigorous	31–150
**THC Users (*n* = 91)**			
Aerobic Exercise			
	Frequency (d/wk)	Intensity	Time (min/wk)
Men (*n* = 35)	4 ± 2	Vigorous*	31–150
Women (*n* = 54)	3 ± 2	Vigorous	31–150
Non-binary (*n* = 2)	5 ± 2	Vigorous	31–150
Resistance Exercise			
Men (*n* = 35)	4 ± 2	Vigorous*	> 150
Women (*n* = 54)	3 ± 1	Vigorous*	31–150
Non-binary (*n* = 2)	3 ± 1	Vigorous	31–150

^*^p < 0.05, significantly different from CBD users

**Table 3:** Exercise modalities being used by cannabis users based on rank

CBD users (*n* = 20)	THC users (*n* = 91)
1. Resistance exercise (80%)	1. Resistance exercise (50%)
2. Running (55%)	2. Running (31%)
3. Hiking (50%)	3. Hiking (31%)
4. Walking (45%)	4. Walking (26%)
5. Yoga (35%)	5. Yoga (21%)
6. Other (30%)	6. Other (10%)
7. Basketball (15%)	7. Basketball (10%)
8. Swimming (10%)	8. Swimming (9%)
9. Martial Arts (15%)	9. Cycling (off road) (7%)
10. Volleyball (5%)	10. Volleyball (7%)
11. Cycling (road) (5%)	11. Golf (6%)
12. Cycling (off road) (5%)	12. Kayak/canoe (6%)
13. Disc Golf (5%)	13. Cycling (road) (5%)
14. Kayak/canoe (5%)	14. Rock climbing (4%)

### Cannabis habits

The majority of participants indicated they had been using CBD for 1–3 years (37%), with participants reporting 1–6 months (10%), 6 months to 1 year (9%), 3–6 years (34%), 6–9 years (10%) and more than 10 years (0%). Thirty-two percent of those CBD users used it seven d/wk, 1–3 times a day (50%), and spent $50–100 per month (21%) on CBD products. Forms of CBD consumption included edibles (30%), vaping (27%), smoking (25%), tinctures (11%), topicals (7%). Negative effects in participants using CBD included felt dehydrated (*n* = 7), lightheadedness (*n* = 3), anxiety (*n* = 3), heart racing (*n* = 2), paranoia (*n* = 1), loss of coordination (*n* = 1), difficulty breathing (*n* = 1), poor reaction time (*n* = 1), other (*n* = 18), and none (*n* = 55).

THC use was the highest at 1–3 years of use (44%), with participants reporting less than 1 month (0%), 1–6 months (7%), 6 months to 1 year (1%), 3–6 years (25%), 6–9 years (5%) and more than 10 years (18%). Fifty-two percent of participants stated that they used THC seven d/wk, 1–3 times a day (76%), and spent $50–100 per month (39%) on products with THC. The forms of THC consumption included smoking (40%), edibles (30%), vaping (26%), tinctures (2%), topicals (1%) and other (1%). Negative effects of THC use included got too high (*n* = 16), too sleepy (*n* = 14), heart racing (*n* = 12), paranoia (*n* = 6), anxiety (*n* = 6), weakness (*n* = 6), difficulty breathing (*n* = 5), laughing uncontrollably (*n* = 3), poor reaction time (*n* = 3), lightheadedness (*n* = 3), poor balance (*n* = 2), enhanced pain (*n* = 1), other (*n* = 16), and none (*n* = 33).

### Recovery from exercise

#### Cannabis for recovery

Twenty-two participants (20%) reported using CBD for recovery from aerobic exercise and 25 participants (23%) reported CBD use to recover from resistance exercise. Sixty-eight participants (61%) reported using THC after aerobic exercise for recovery. Similarly, 67 participants (60%) reported using THC after resistance exercise for recovery. When participants were asked, ‘Do you feel that cannabis in the form of CBD aids in your recovery?’ 93% stated ‘yes’ while 7% stated, “I’m not sure.’ When asked, ‘Do you feel that THC aids in your recovery?’, 87% of participants stated, ‘yes’ while 13% stated, ‘I’m not sure’. No participant answered, ‘no’, for either of these questions. All of the participants felt that use of cannabis was low risk regarding health outcomes.

Supplements used by the participants are shown in Table 4. The supplements highlighted by the participants are similar between those that use CBD and those that use THC. The supplements used by the participants are focused on performance (caffeine, for example) and recovery (creatine, protein powder). However, there were a small handful of THC users that listed steroids as a supplement, which was not noted in those using CBD.

**Table 4:** Supplements being taken by cannabis users based on rank

CBD users (*n* = 20)	THC users (*n* = 91)
1. Protein Powder (30%)	1. Protein Powder (21%)
2. Creatine (25%)	2. Caffeine (18%)
3. Caffeine (25%)	3. Creatine (14%)
4. BCAA (15%)	4. Fish oil (10%)
5. Beta Alanine (10%)	5. Beta alanine (8%)
6. Fish Oil (10%)	6. Probiotics (8%)
7. Glutamine (5%)	7. BCAA (7%)
8. Glucosamine (5%)	8. Herbal Supplements (2%)
9. Others (5%)	9. Steroids (2%)

Motives for CBD use demonstrated that participants primarily used it to assist with sleep (3.89) and to relax (3.89). Other reasons for CBD use were because it gives a pleasant feeling (3.37), and to alleviate pain (3.05). Motives for THC use was primarily to relax (4.40). Other notable reasons included because it gives a pleasant feeling (4.17), is fun (3.79), to assist with sleep (3.81), to alleviate pain (3.10), to help alleviate depression or nervousness (3.29), and to cheer up when in a bad mood (3.21). There were no significant correlations between motivations for cannabis use and self-reported recovery.

## Discussion

The primary aim of this study was to determine if individuals are using CBD and/or THC to assist recovery from exercise and if they believed cannabis to be an effective recovery modality. The data from the present study suggest that individuals actively use CBD and/or THC as part of their recovery. In addition, our participants stated that they felt that cannabis assisted with their recovery from both aerobic and resistance exercise.

Studies have shown cannabis, CBD or THC, has a minimal effect on performance (Renaud and Cormier 1986; Lisano et al. 2019). Interestingly, in the present study, men that were CBD users had a higher intensity of aerobic and resistance exercise compared to those that used THC only. For the women CBD users, the intensity was higher for aerobic activity compared to women that were using THC. These self-reported measures of exercise intensity provide insight into the perception of those using cannabis that are exercising regularly. In addition, it is possible that due to the reduced exercise intensity in THC users that recovery seemed more efficient due to reductions in intensity. Regardless, cannabis has been suggested to assist with pain management, inflammation, and sleep, all of which are essential for recovery. YorkWilliams et al. (2019) reported 77.6% (481/620) of their participants agreed or strongly agreed that THC use enhanced recovery from exercise, while 16.3% (101/620) were neutral and 6.1% (38/620) disagreed or disagreed strongly (YorkWilliams et al. 2019). This is in agreement, to some degree, with findings from the present study. In the present study, 93% stated they believed CBD use improved recovery and 87% of participants stated they believed THC use aided in recovery. The difference between the present study and the work by YorkWilliams et al. (2019) is the present study demonstrated that many participants reported being unsure if CBD or THC aided in recovery, and none that stated ‘no’. There are limited data on exercise recovery and cannabis (YorkWilliams et al. 2019). Interestingly, Isenmann et al. (2021) had participants consume CBD and then perform an intense acute bout of resistance exercise. Twenty-four hours, 48 h, and 72 h following the intensive acute bout of resistance exercise, participants completed a one-repetition maximum strength test (1RM) (Isenmann et al. 2021). Isenmann et al. (2021) noted that following consumption of CBD that the 1RM was maintained, and slightly higher, then the initial measurement at 72 h (Isenmann et al. 2021). Therefore, it is important to understand how cannabis, CBD or THC, plays in role in recovery, particularly pain management, inflammation, and sleep.

One of the primary purported roles of CBD or THC is pain management (Ajrawat et al. 2022; Cuttler et al. 2022). The present study demonstrated that participants used CBD and THC as a means to reduce pain, which is in agreement with our hypothesis. While numerous studies have demonstrated CBD or THC use may have a profound effect on chronic pain (Cuttler et al. 2022), the data are not conclusive (Ajrawat et al. 2022). Regarding acute pain management and muscle soreness, the data are sparse. A study by Kasper et al. (2020) demonstrated that in professional rugby players, (133/517) 26% used CBD. While 80% of athletes using CBD felt it would improve recovery and sleep, only 40% reported benefits in these areas. Additionally, only 15% of athletes reported pain relief with CBD consumption (Kasper et al. 2020). Deckey et al. (2022) reported that 19% of respondents (152/823) reported using CBD prior to an initial evaluation of a sports medicine injury (Deckey et al. 2022). Of those individuals, 30% (46/152) were also using THC (Deckey et al. 2022). Cuttler et al. (2022) reported reduced acute pain severity by 42–49% when cannabis was inhaled (Cuttler et al. 2022). Collectively, the data suggests that cannabis is a strong mediator of pain, but the data are not conclusive.

Multiple studies to date have detailed immune responses with CBD or THC consumption surrounding exercise (Sahinovic et al. 2022). Sahinovic et al. (2022) had participants consume CBD prior to maximal aerobic exercise and reported possible reductions in inflammatory cytokines TNF- α and IL-6, with no changes in creatine kinase (Sahinovic et al. 2022). Isenmann et al. (2021) reported that 72 h after an intense acute bout of resistance exercise there was a diminished increase in myoglobin when CBD was administered prior to the exercise with no differences in creatine kinase, both markers of skeletal muscle damage (Isenmann et al. 2021). Accordingly, more substantial evidence exists in support of CBD use or CBD use in combination with THC use attenuating inflammation at a greater magnitude when compared to THC use alone.

Sleep is an important aspect of recovering from exercise (Kasper et al. 2020; Shannon et al. 2019). In the present study, one of the primary motives for using CBD and/or THC was to improve sleep, supporting our hypothesis. Data have suggested that CBD or THC use may have sleep enhancing effects (Kasper et al. 2020), but this is inconclusive (Shannon et al. 2019). Shannon et al. (2019), using the Pittsburgh Sleep Quality Index, demonstrated an improvement of sleep in 66.7% (48/72) of their participants after one month of dosing participants with CBD (25 mg/d—175 mg/d), while 25% (18/72) experienced lesser sleep quality (Shannon et al. 2019). Two months of taking CBD resulted in 56.1% (40/72) having improved sleep and 26.8% (19/72) experiencing worse sleep. A review of literature by Babson et al. (2017) suggested that acute THC use improves sleep while chronic THC use has been suggested to worsen sleep (Babson et al. 2017). Sahinovic et al. (2022) reported no differences in sleep 24 h after consuming CBD prior to performing submaximal aerobic exercise (Sahinovic et al. 2022). In addition, chronic cannabis use for sleep purposes may inherently increase cannabis dependence such that once habituation to THC is achieved, more THC is needed resulting in altered patterns of use, and ultimately less sleep (Babson et al. 2017).

While the present study demonstrates that individuals are using cannabis to recover from exercise, there are a litany of other modalities that are being used in conjunction as well. However, evidence varies heavily between modalities. Studies have demonstrated that implementation of a static stretching protocol following a bout of exercise does not improve perceived muscle soreness, enhance exercise performance in a subsequent exercise bout, or reduce inflammatory markers in both recreationally active and athletic populations (Bonfim et al. 2010; Cesar et al. 2021). Few studies have evaluated the effect of hot tub or sauna interventions on exercise recovery. Overall, no reduction in perceived muscle soreness or enhancement in exercise performance has been observed with hot tub interventions, while a post-exercise sauna intervention has induced performance decrements (Pournot et al. 2011; Skorski et al. 2019). Conversely, both pre- and post-exercise foam rolling interventions have stimulated more rapid recovery in sprint or strength performance following a bout of exercise, while also reducing levels of perceived muscle soreness (D'Amico and Gillis 2019). Cupping therapy has been suggested to reduce muscle fatigue without mediating adverse effects on the skeletal muscle (Lowe 2017) and has been shown to improve local blood flow (Hou et al. 2021), alleviate muscle pain (Kim et al. 2012), and reduce muscle stiffness (Jan et al. 2021). Electrical stimulation has been demonstrated to be a safe and effective modality to reduce muscle soreness (Babault et al. 2011), but the effects on inflammation are unclear (Lambernd et al. 2012). Compression garments have also been observed to minorly attenuate post-exercise muscle soreness and perceived levels of fatigue but induce no changes in indicators of inflammation such as creatine-kinase or CRP (Pruscino et al. 2013). Cryotherapy, exposure of the body to freezing temperatures (-110 to -150ºC for 2–3 min), has been shown to increase the anti-inflammatory cytokine IL-10, and decrease proinflammatory cytokine IL-2 and chemokine IL-8 (Banfi et al. 2009). Contrast water therapy, or alternating hot- and cold-water immersion, has demonstrated the ability to enhance perceived recovery, perceived muscle soreness, exercise performance, and has reduced creatine kinase 24- and 48-h post-exercise (Coffey et al. 2004). Cold-water immersion has been well-established to attenuate the production of pro-inflammatory cytokines such as TNF-α and IL-1β, while also increasing IL-10, a key mediator of the anti-inflammatory response (Eimonte et al. 2021). Additionally, multiple investigations have reported enhancements in recovery of exercise performance and reduced feelings of perceived muscle soreness with cold water immersion (Ingram et al. 2009). There are also various supplements that are used to speed recovery such as branch chain amino acids, creatine monohydrate, and protein supplementation, to name a few. Collectively, there are modalities and supplements being used for their ability to maintain exercise or athletic performance in the days or hours following strenuous activity and to mitigate muscle soreness. Consequently, there is substantial overlap between the mechanistic benefits of cannabis for recovery and those of these aforementioned modalities and supplements.

The present study is not without limitations. The legality of cannabis across states generates an inherent issue regarding legality of cannabis use across individuals that participated in the present study. While the present study collected data on which states individuals reside, it was not the purpose of the present study to delineate which users were following state laws regarding cannabis use. The purpose of the present was exploratory in nature, such that connections between cannabis use and recovery from exercise are anecdotal. In addition, the data collected are self-reported. The sample size was also small, which may limit the ability to draw conclusions. Lastly, the majority of the participants were from the Midwest region of the United States, which has limited access to cannabis medically or recreationally.

## Conclusions

The effects of cannabis on recovery are not well understood and there are limited data directly examining the relationship. However, recovery from exercise is multi-faceted and complex. CBD or THC use have been suggested to play a role in pain management, inflammation, and sleep, which are hallmarks for recovery from exercise. While data are lacking, it is clear that individuals are using cannabis and believe cannabis to have a positive effect on recovery from exercise. The present study demonstrated that in addition to more traditional recovery methods, cannabis is used as an ergogenic recovery aid by individuals that exercise regularly. Regardless, the present study was exploratory in nature, and it is clear that more data are needed regarding cannabis, CBD and THC, on recovery from exercise.

### Supplementary Information

**Additional file 1. **Cannabis survey.

## Data Availability

The datasets used and/or analyzed during the current study are available from the corresponding author on reasonable request.

## Abbreviations

CBD: Cannabidiol
THC: Delta-9-tetrohydrocannabidiol
CB1: Cannabinoid type 1
CB2: Cannabinoid type 2
TNF: Tumor necrosis factor
IL: Interleukin
CRP: C-reactive protein
