# Supplementary material for: Cannabis use for exercise recovery in trained individuals: a survey study
Source: J Cannabis Res. 2023 Aug 5;5:32. doi: 10.1186/s42238-023-00198-5 (PMC10403841; doi:10.1186/s42238-023-00198-5)
Supplement: Supplementary file 1 — Additional file 1. Cannabis survey. [file 42238_2023_198_MOESM1_ESM.pdf]

Cannabis Survey

Start of Block: Informed Consent

Informed Consent **Informed Consent to Participate in a Research Study**

You are being invited to participate in a study that will provide researchers and professionals with important information regarding health behaviors and attitudes. Specifically, the survey asks about your cannabis consumption, perception of cannabis, and physical activity behavior.

Participation is voluntary, refusal to take part in the study involves no penalty, and you may withdraw from the study at any time without penalty or cost. The study is being conducted by Emily Erb, M.S., Stacie Humm, M.S. and Dr. J. Derek Kingsley of Kent State University, and it has been approved by the Kent State University Institutional Review Board.

Participation involves completing a brief 10-15 minute online survey. Participation is completely anonymous – no identifiable data are being collected. There are no anticipated risks associated with your participation in this study beyond those encountered in everyday life. Participants must be at least 18 years of age. There are no direct benefits of participating in the study. However, what we learn from the study may help us to better understand health and well-being and how cannabis is consumed and used in a health and fitness setting.

<u>All information gathered will be kept anonymous and will not be tracible to you.</u>

If you have any questions or comments about the survey or are curious about the results, please contact Emily Erb by email (eerb4@kent.edu), Stacie Humm (shumm2@kent.edu), or Dr. J. Derek Kingsley by email (jkingsle@kent.edu), or the Kent State University Institutional Review Board, at (330) 672-2704. The Survey begins on the next page. Beginning the survey implies that you have read this consent form, voluntarily agree to participate in this study, and are at least 18 years of age. Thank you for your participation, it is much appreciated.

Sincerely,

Emily Erb, M.S. and Stacie Humm, M.S.
Doctoral students

J. Derek Kingsley, Ph.D. FACSM
Associate Professor and Program Coordinator
Exercise Science and Exercise Physiology
Kent State University

○ I have read the informed consent, am at least 18 years of age, and volunteer to take this survey. (1)

○ I am not at least 18 years of age, and/or do not wish to volunteer to take this survey. (2)

Skip To: End of Survey If Informed Consent to Participate in a Research Study You are being invited to participate in a stu... = I am not at least 18 years of age, and/or do not wish to volunteer to take this survey.

End of Block: Informed Consent

Start of Block: Cannabis Habits

Q51 The first section of questions aim to gather information about your cannabis habits. These include questions about both cannabis in the form of CBD and cannabis with THC. Please read each question carefully and respond accordingly.

- ○ Proceed to questions (1)

End of Block: Cannabis Habits

Start of Block: Cannabis Habits

1 Do you regularly use cannabis in the form of CBD?

- ○ Yes (1)
- ○ No (2)
- ○ Prefer not to answer (3)

Skip To: 2 If Do you regularly use cannabis in the form of CBD? != Yes

Page Break

1a For how long have you been using cannabis in the form of CBD?

- ○ Less than 1 month (1)
- ○ 1 - 6 months (2)
- ○ 6 months - 1 year (3)
- ○ 1 - 3 years (4)
- ○ 3 - 6 years (5)
- ○ 6 - 9 years (6)
- ○ 10 or more years (7)

1b * On average, on how many days per week do you use cannabis in the form of CBD?

- ○ 1 (1)
- ○ 2 (2)
- ○ 3 (3)
- ○ 4 (4)
- ○ 5 (5)
- ○ 6 (6)
- ○ 7 (7)

1c On average, how many times per day do you use cannabis in the form of CBD?

- Less than 1 (1)
- 1-3 (2)
- 4-6 (3)
- 7-9 (4)
- 10 or more (5)

1d On average, how much money do you spend on cannabis products in the form of CBD each month?

- $0 per month (1)
- $50 or less per month (2)
- $51 - $100 per month (3)
- $101 - $150 per month (4)
- More than $150 per month (5)

1e What is your preferred method of cannabis in the form of CBD use (choose all that apply)?

- ☐ Smoking (1)
- ☐ Vaping (2)
- ☐ Edibles (3)
- ☐ Tinctures (oil) (4)
- ☐ Topicals (5)
- ☐ Other (6)

Page Break

2 Do you regularly use cannabis with THC?

- Yes (1)
- No (2)
- Prefer not to answer (3)

Skip To: End of Block If Do you regularly use cannabis with THC? != Yes

Page Break

2a For how long have you been using cannabis with THC?

- Less than 1 month (1)
- 1 - 6 months (2)
- 6 months - 1 year (3)
- 1 - 3 years (4)
- 3 - 6 years (5)
- 6 - 9 years (6)
- 10 or more years (7)

2b On average, on how many days per week do you use cannabis with THC?

- 1 (2)
- 2 (3)
- 3 (4)
- 4 (5)
- 5 (6)
- 6 (7)
- 7 (8)

2c On average, how many times per day do you use cannabis with THC?

- 1 - 3 (1)
- 4 - 6 (2)
- 7 - 9 (3)
- 10 or more (4)

2d On average, how much money do you spend on cannabis products with THC each month?

- $0 per month (1)
- Less than $50 per month (2)
- $50 - $100 per month (3)
- $101 - $150 per month (4)
- More than $150 per month (5)

2e What is your preferred method of cannabis with THC use (choose all that apply)?

- Smoking (1)
- Vaping (2)
- Edibles (3)
- Tinctures (oil) (4)
- Topicals (5)
- Other (6)

End of Block: Cannabis Habits

Start of Block: Exercise Habits

Q50 The next series of questions will inquire about your exercise habits. Please read each question carefully and respond accordingly.

○ Proceed to questions (1)

End of Block: Exercise Habits

Start of Block: Exercise Habits

1 Do you currently exercise?

○ Yes (1)

○ No (2)

Skip To: End of Survey If Do you currently exercise? = No

Page Break

2 For how long have you been exercising?

- Less than 1 year (1)
- 1 - 3 years (2)
- 3 - 5 years (3)
- More than 5 years (4)

---

3 During the average week, do you exercise with a team?

- Yes (1)
- No (2)

---

4 On average, how many minutes per week do you spend exercising?

- Less than 30 minutes (1)
- 30 - 150 minutes (2)
- 151 - 300 minutes (3)
- More than 300 minutes (4)

Page Break

5 What are your main modalities of exercise (choose all that apply)?

- ☐ Hiking (1)
- ☐ Yoga (2)
- ☐ Resistance exercise (3)
- ☐ Swimming (4)
- ☐ Running (5)
- ☐ Cycling (road) (6)
- ☐ Cycling (off road) (7)
- ☐ Bowling (8)
- ☐ Basketball (9)
- ☐ Volleyball (10)
- ☐ Baseball / Softball (11)
- ☐ Golf (12)
- ☐ Disc golf (13)
- ☐ Skiing (winter) (14)
- ☐ Skiing (summer) (15)
- ☐ Snowboarding (16)
- ☐ Cross country skiing (17)
- ☐ Archery (18)

- ☐ Hockey (19)
- ☐ Equestrian (20)
- ☐ Sailing (21)
- ☐ Martial arts (22)
- ☐ Rock climbing (23)
- ☐ Kayak / Canoe (24)
- ☐ Paddleboarding (25)
- ☐ Skateboarding (26)
- ☐ Walking (27)
- ☐ Other (29)

Page Break

6 On average, do you engage in aerobic exercise?

- ◯ Yes (1)
- ◯ No (2)

Skip To: 10 If On average, do you engage in aerobic exercise? = No

Page Break

7 On average, what is the intensity of your aerobic exercise workouts?

- Max efforts (1)
- Very hard (2)
- Vigorous (3)
- Moderate (4)
- Light (5)
- Very light (6)

---

8 During the average week, on how many days do you engage in aerobic exercise?

- 1 (2)
- 2 (3)
- 3 (4)
- 4 (5)
- 5 (6)
- 6 (7)
- 7 (8)

---

9 On average, how many minutes per week do you spend aerobically exercising?

- ○ Less than 30 minutes (1)
- ○ 31 - 150 minutes (2)
- ○ 151 - 300 minutes (3)
- ○ More than 300 minutes (4)

Page Break

10 On average, do you engage in resistance exercise?

- ○ Yes (1)
- ○ No (2)

Skip To: 14 If On average, do you engage in resistance exercise? = No

Page Break

11 On average, what is the intensity of your resistance exercise workouts?

- Max effort (1)
- Very hard (2)
- Vigorous (3)
- Moderate (4)
- Light (5)
- Very light (6)

---

12 During the average week, on how many days do you engage in resistance exercise?

- 1 (2)
- 2 (3)
- 3 (4)
- 4 (5)
- 5 (6)
- 6 (7)
- 7 (8)

---

13 On average, how many minutes per week do you spend resistance exercising?

- ○ Less than 30 minutes (1)
- ○ 30 - 150 minutes (2)
- ○ 151 - 300 minutes (3)
- ○ More than 300 minutes (4)

Page Break

14 Are you currently using supplements to enhance your exercise performance?

- ○ Yes (1)
- ○ No (2)

Skip To: 15 If Are you currently using supplements to enhance your exercise performance? = No

Page Break

14a Please select all supplements you currently use to enhance your exercise performance.

- ☐ Creatine (1)
- ☐ HMB (2)
- ☐ Nitric oxide (3)
- ☐ Protein powder (4)
- ☐ Caffeine (5)
- ☐ Steroids (6)
- ☐ Glutamine (7)
- ☐ BCAA (8)
- ☐ Beta alanine (9)
- ☐ DHEA (10)
- ☐ Iron (11)
- ☐ Quercin (12)
- ☐ Tart cherry juice (13)
- ☐ Green tea extract (14)
- ☐ Glucosamine (15)
- ☐ Fish oil (16)
- ☐ Probiotics (17)
- ☐ Herbal supplements (18)

☐ Other (19)

Page Break

15 Do you use exercise recovery methods that do not involve cannabis use (check all that apply)?

- ▢ Sauna (1)
- ▢ Stretching (2)
- ▢ Massage (3)
- ▢ Foam rolling (4)
- ▢ Hot tub (13)
- ▢ Cupping (5)
- ▢ Electrical stimulation (6)
- ▢ Compression garments (7)
- ▢ Ice baths (8)
- ▢ Cryotherapy (9)
- ▢ Contrast water bath (10)
- ▢ Supplements other than cannabis (11)
- ▢ Other exercise recovery methods (12)
- ▢ No exercise recovery methods (14)

End of Block: Exercise Habits

Start of Block: Exercise and Cannabis

Q52 The following questions aim to gather information on your exercise habits in relation to cannabis use in the form of CBD. Please read each question carefully and respond accordingly.

○ Proceed to questions (1)

End of Block: Exercise and Cannabis

Start of Block: Exercise Habits and CBD

1 Do you use cannabis before, during, or after exercise in the form of CBD?

○ Yes (1)

○ No (2)

○ Prefer not answer (3)

Skip To: 1m If Do you use cannabis before, during, or after exercise in the form of CBD? != Yes

Page Break

1a On average, do you use cannabis in the form of CBD prior to engaging in aerobic exercise?

- ○ Yes (1)
- ○ No (2)

Skip To: 1c If On average, do you use cannabis in the form of CBD prior to engaging in aerobic exercise? = No

Page Break

1b On average, how frequently do you use cannabis in the form of CBD <u>prior to</u> aerobic exercise?

- ○ 100% of the time (1)
- ○ 75% of the time (2)
- ○ 50% of the time (3)
- ○ 25% of the time (4)
- ○ Less than 25% of the time (5)

Page Break

1c On average, do you use cannabis in the form of CBD prior to resistance exercise?

○ Yes (1)

○ No (2)

Skip To: 1e If On average, do you use cannabis in the form of CBD prior to resistance exercise? = No

Page Break

1d On average, how frequently do you use cannabis in the form of CBD prior to resistance exercise?

- 100% of the time (1)
- 75% of the time (2)
- 50% of the time (3)
- 25% of the time (4)
- Less than 25% of the time (5)

Page Break

1e On average, do you use cannabis in the form of CBD <u>during</u> aerobic exercise?

○ Yes (1)

○ No (2)

Skip To: 1g If On average, do you use cannabis in the form of CBD during aerobic exercise? = No

Page Break

1f On average, how frequently do you use cannabis in the form of CBD <u>during</u> aerobic exercise?

○ 100% of the time (1)

○ 75% of the time (2)

○ 50% of the time (3)

○ 25% of the time (4)

○ Less than 25% of the time (5)

Page Break

1g On average, do you use cannabis in the form of CBD <u>during</u> in resistance exercise?

- ○ Yes (1)
- ○ No (2)

Skip To: 1i If On average, do you use cannabis in the form of CBD during in resistance exercise? = No

Page Break

1h On average, how frequently do you use cannabis in the form of CBD <u>during</u> resistance exercise?

- 100% of the time (1)
- 75% of the time (2)
- 50% of the time (3)
- 25% of the time (4)
- Less than 25% of the time (5)

Page Break

1i On average, do you use cannabis in the form of CBD after engaging in aerobic exercise?

- ○ Yes (1)
- ○ No (2)

Skip To: 1k If On average, do you use cannabis in the form of CBD after engaging in aerobic exercise? = No

Page Break

1j On average, how frequently do you use cannabis in the form of CBD after engaging in aerobic exercise?

- 100% of the time (1)
- 75% of the time (2)
- 50% of the time (3)
- 25% of the time (4)
- Less than 25% of the time (5)

Page Break

1k On average, do you use cannabis in the form of CBD <u>after</u> engaging in resistance exercise?

○ Yes (1)

○ No (2)

Skip To: 1m If On average, do you use cannabis in the form of CBD after engaging in resistance exercise? = No

Page Break

1l On average, how frequently do you use cannabis in the form of CBD after resistance exercise?

- 100% of the time (1)
- 75% of the time (2)
- 50% of the time (3)
- 25% of the time (4)
- Less than 25% of the time (5)

Page Break

1m Do you use cannabis in the form of CBD to assist recovery from exercise?

- ○ Yes (1)
- ○ No (2)

Skip To: 1p If Do you use cannabis in the form of CBD to assist recovery from exercise? = No

Page Break

1n How often do you use cannabis in the form of CBD for exercise recovery?

- 100% of the time (1)
- 75% of the time (2)
- 50% of the time (3)
- 25% of the time (4)
- Less than 25% of the time (5)

Page Break

1o Do you feel that cannabis with CBD aids in your recovery?

- ○ Yes (1)
- ○ No (2)
- ○ I'm not sure (3)

Page Break

1p Do you feel that exercise before, during, or after the use of cannabis in the form of CBD is different than exercise without cannabis in the form of CBD (choose all that apply)?

- ▢ Yes, more focused with CBD (1)
- ▢ Yes, more satisfied with CBD (2)
- ▢ Yes, more productive with CBD (3)
- ▢ Yes, better form with CBD (4)
- ▢ No difference (5)
- ▢ Yes, less focused with CBD (6)
- ▢ Yes, less satisfied with CBD (7)
- ▢ Yes, less productive with CBD (8)
- ▢ Yes, poorer form with CBD (9)
- ▢ I'm not sure (11)

Page Break

1q Have you had any unanticipated/undesired experiences while exercising before, during, or after the use of cannabis in the form of CBD (select all that apply)?

- [ ] Got too high to be effective (1)
- [ ] Heart racing (2)
- [ ] Lightheadedness (3)
- [ ] Paranoia (4)
- [ ] Anxiety (5)
- [ ] Weakness (6)
- [ ] Loss of coordination (7)
- [ ] Difficulty breathing (8)
- [ ] Poor reaction time (9)
- [ ] Poor balance (10)
- [ ] Hallucinations (11)
- [ ] Passed out (12)
- [ ] Enhanced pain (13)
- [ ] Injury (14)
- [ ] Too sleepy (15)
- [ ] Laughing uncontrollably (16)
- [ ] Nausea (17)

☐ Felt dehydrated (18)

☐ Other (19)

☐ None (20)

End of Block: Exercise Habits and CBD

Start of Block: Exercise Habits and THC

Q72 The following questions aim to gather information on your exercise habits in relation to cannabis use with THC. Please read each question carefully and respond accordingly.

○ Proceed to questions (1)

End of Block: Exercise Habits and THC

Start of Block: Exercise Habits and THC

2 Do you use cannabis with THC before, during, or after exercise?

○ Yes (1)

○ No (2)

○ Prefer not answer (3)

Skip To: 2m If Do you use cannabis with THC before, during, or after exercise? != Yes

Page Break

2a On average, do you use cannabis with THC prior to engaging in aerobic exercise?

- ○ Yes (1)
- ○ No (2)

Skip To: 2b If On average, do you use cannabis with THC prior to engaging in aerobic exercise? = Yes

Skip To: 2c If On average, do you use cannabis with THC prior to engaging in aerobic exercise? = No

Page Break

2b On average, how frequently do you use cannabis with THC prior to aerobic exercise?

- 100% of the time (1)
- 75% of the time (2)
- 50% of the time (3)
- 25% of the time (4)
- Less than 25% of the time (5)

Page Break

2c On average, do you use cannabis with THC prior to resistance exercise?

- ○ Yes (1)
- ○ No (2)

Skip To: 2e If On average, do you use cannabis with THC prior to resistance exercise? = No

Page Break

2d On average, how frequently do you use cannabis with THC prior to resistance exercise?

- 100% of the time (1)
- 75% of the time (2)
- 50% of the time (3)
- 25% of the time (4)
- Less than 25% of the time (5)

Page Break

2e On average, do you use cannabis with THC during aerobic exercise?

- ○ Yes (1)
- ○ No (2)

Page Break

2f On average, how frequently do you use cannabis with THC during aerobic exercise?

- ○ 100% of the time (1)
- ○ 75% of the time (2)
- ○ 50% of the time (3)
- ○ 25% of the time (4)
- ○ Less than 25% of the time (5)

Page Break

2g On average, do you use cannabis with THC during resistance exercise?

- Yes (1)
- No (2)

Skip To: 2i If On average, do you use cannabis with THC during resistance exercise? = No

Page Break

2h On average, how frequently do you use cannabis with THC <u>during</u> resistance exercise?

- ○ 100% of the time (1)
- ○ 75% of the time (2)
- ○ 50% of the time (3)
- ○ 25% of the time (4)
- ○ Less than 25% of the time (5)

Page Break

2i On average, do you use cannabis with THC after engaging in aerobic exercise?

- ◯ Yes (1)
- ◯ No (2)

Skip To: 2k If On average, do you use cannabis with THC after engaging in aerobic exercise? = No

Page Break

2j On average, how frequently do you use cannabis with THC after aerobic exercise?

- 100% of the time (1)
- 75% of the time (2)
- 50% of the time (3)
- 25% of the time (4)
- Less than 25% of the time (5)

Page Break

2k On average, do you use cannabis with THC <u>after</u> engaging in resistance exercise?

○ Yes (1)

○ No (2)

Skip To: 2m If On average, do you use cannabis with THC after engaging in resistance exercise? = No

Page Break

2l On average, how frequently do you use cannabis with THC after resistance exercise?

- 100% of the time (1)
- 75% of the time (2)
- 50% of the time (3)
- 25% of the time (4)
- Less than 25% of the time (5)

Page Break

2m Do you use cannabis with THC to assist recovery from exercise?

- ○ Yes (1)
- ○ No (2)

Skip To: 2p If Do you use cannabis with THC to assist recovery from exercise? = No

Page Break

2n How often do you use cannabis with THC for exercise recovery?

- ◯ 100% of the time (1)
- ◯ 75% of the time (2)
- ◯ 50% of the time (3)
- ◯ 25% of the time (4)
- ◯ Less than 25% of the time (5)

Page Break

2o Do you feel that cannabis with THC aids in your recovery?

- Yes (1)
- No (2)
- I'm not sure (3)

Page Break

2p Do you feel that exercise before, during, or after the use of cannabis in the form of THC is different than exercise without cannabis with THC (choose all that apply)?

- ☐ Yes, more focused with THC (1)
- ☐ Yes, more satisfied with THC (2)
- ☐ Yes, more productive with THC (3)
- ☐ Yes, better form with THC (4)
- ☐ No difference (5)
- ☐ Yes, less focused with THC (7)
- ☐ Yes, less satisfied with THC (8)
- ☐ Yes, less productive with THC (9)
- ☐ Yes, poorer form with THC (10)
- ☐ I'm not sure (11)

Page Break

2q Have you had any unanticipated/undesired experiences while exercising before, during, or after the use of cannabis with THC (select all that apply)?

- ☐ Got too high to be effective (1)
- ☐ Heart racing (2)
- ☐ Lightheadedness (3)
- ☐ Paranoia (4)
- ☐ Anxiety (5)
- ☐ Weakness (6)
- ☐ Loss of coordination (7)
- ☐ Difficulty breathing (8)
- ☐ Poor reaction time (9)
- ☐ Poor balance (10)
- ☐ Hallucinations (11)
- ☐ Passed out (12)
- ☐ Enhanced pain (13)
- ☐ Injury (14)
- ☐ Too sleepy (15)
- ☐ Laughing uncontrollably (16)
- ☐ Nausea (17)

☐ Felt dehydrated (18)

☐ Other (19)

☐ None (20)

End of Block: Exercise Habits and THC

Start of Block: CBD Motives

Q73 Next, you will be asked about your potential motives behind the use of cannabis in the form of CBD. Please read each question carefully and respond accordingly.

○ Proceed to questions (1)

Page Break

1 Do you ever consume cannabis in the form of CBD?

○ Yes (1)

○ No (2)

Skip To: End of Block If Do you ever consume cannabis in the form of CBD? = No

Page Break

2 Please indicate how often you consume cannabis in the form of CBD for the following reasons:

	Never / Almost never (1)	Rarely (2)	Sometimes (3)	Often (4)	Always / Almost always (5)
To forget my worries (1)					
Because my friends pressure me to (2)					
Because it helps me enjoy a party (3)					
Because it helps me when I feel depressed or nervous (4)					
To be sociable (5)					
To cheer me up when I am in a bad mood (6)					
Because I like the feeling (7)					
So that others won't kid me about not using CBD (8)					
Because it is exciting (9)					
To get high (10)					
Because it makes social gatherings					

more fun (11)					
To fit in with the group I like (12)	○	○	○	○	○
Because it gives me a pleasant feeling (13)	○	○	○	○	○
Because it improves parties and celebrations (14)	○	○	○	○	○
Because I feel more self-confident and sure of myself (15)	○	○	○	○	○
To relax (16)	○	○	○	○	○
To forget about my problems (17)	○	○	○	○	○
Because it is fun (18)	○	○	○	○	○
To be liked (19)	○	○	○	○	○
So I won't feel left out (20)	○	○	○	○	○
To know myself better (21)	○	○	○	○	○
Because it helps me to be more creative and original (22)	○	○	○	○	○
To understand things differently	○	○	○	○	○

(23)					
To expand my awareness (24)	○	○	○	○	○
Because it's relatively low risk (25)	○	○	○	○	○
To assist with sleep (26)	○	○	○	○	○
To alleviate loss of appetite (27)	○	○	○	○	○
To improve exercise performance (28)	○	○	○	○	○
To alleviate pain (29)	○	○	○	○	○
For reasons not previously mentioned (30)	○	○	○	○	○

Page Break

3 Do you think consuming cannabis in the form of CBD is relatively low risk?

- ○ Yes (1)
- ○ No (2)

---

4 Do you feel that cannabis use in the form of CBD will have health consequences such as higher risk for lung infections, weakened immune system, temporary hallucinations, and paranoia?

- ○ Yes (1)
- ○ No (2)

Page Break

Display This Question:

If Do you feel that cannabis use in the form of CBD will have health consequences such as higher ris... = Yes

4a Please indicate if you think the following forms of CBD will have any of the following long-term health consequences:

	Lung infections (1)	Weakened immune system (2)	Temporary hallucinations (3)	Paranoia (4)	Other (5)
Smoking (1)	▢	▢	▢	▢	▢
Vaping (2)	▢	▢	▢	▢	▢
Edibles (3)	▢	▢	▢	▢	▢
Tinctures (oil) (4)	▢	▢	▢	▢	▢
Topicals (5)	▢	▢	▢	▢	▢
Other (6)	▢	▢	▢	▢	▢

Page Break

5 Do you feel that cannabis in the form of CBD has short-term consequences such as memory loss, loss of coordination, anxiety, and altered senses?

- ◯ Yes (1)
- ◯ No (2)

End of Block: CBD Motives

Start of Block: THC Motives

Q80 Next, you will be asked about your potential motives behind the use of cannabis with THC. Please read each question carefully and respond accordingly.

- ◯ Proceed to questions (1)

Page Break

1 Do you ever consume cannabis with THC?

- Yes (1)
- No (2)

Skip To: End of Block If Do you ever consume cannabis with THC? = No

Page Break

2 Please indicate how often you consume cannabis with THC for the following reasons:

	Never / Almost never (1)	Rarely (2)	Sometimes (3)	Often (4)	Always / Almost always (5)
To forget my worries (1)	○	○	○	○	○
Because my friends pressure me to (2)	○	○	○	○	○
Because it helps me enjoy a party (3)	○	○	○	○	○
Because it helps me when I feel depressed or nervous (4)	○	○	○	○	○
To be sociable (5)	○	○	○	○	○
To cheer me up when I am in a bad mood (6)	○	○	○	○	○
Because I like the feeling (7)	○	○	○	○	○
So that others won't kid me about not using THC (8)	○	○	○	○	○
Because it is exciting (9)	○	○	○	○	○
To get high (10)	○	○	○	○	○
Because it makes social gatherings more fun (11)	○	○	○	○	○

To fit in with the group I like (12)	○	○	○	○	○
Because it gives me a pleasant feeling (13)	○	○	○	○	○
Because it improves parties and celebrations (14)	○	○	○	○	○
Because I feel more self-confident and sure of myself (15)	○	○	○	○	○
To relax (16)	○	○	○	○	○
To forget about my problems (17)	○	○	○	○	○
Because it is fun (18)	○	○	○	○	○
To be liked (19)	○	○	○	○	○
So I won't feel left out (20)	○	○	○	○	○
To know myself better (21)	○	○	○	○	○
Because it helps me to be more creative and original (22)	○	○	○	○	○
To understand things differently (23)	○	○	○	○	○

To expand my awareness (24)	◯	◯	◯	◯	◯
Because it's relatively low risk (25)	◯	◯	◯	◯	◯
To assist with sleep (26)	◯	◯	◯	◯	◯
To alleviate loss of appetite (27)	◯	◯	◯	◯	◯
To improve exercise performance (28)	◯	◯	◯	◯	◯
To alleviate pain (29)	◯	◯	◯	◯	◯
For reasons not previously mentioned (30)	◯	◯	◯	◯	◯

Page Break

3 Do you think consuming cannabis with THC is relatively low risk?

○ Yes (1)

○ No (2)

---

4 Do you feel that cannabis use with THC will have health consequences such as higher risk for lung infections, weakened immune system, temporary hallucinations, and paranoia?

○ Yes (1)

○ No (2)

Skip To: 5 If Do you feel that cannabis use with THC will have health consequences such as higher risk for lung... = No

---

Page Break

4a Please indicate if you think the following forms of cannabis with THC will have any of the following health consequences:

	Lung infections (1)	Weakened immune system (2)	Temporary hallucinations (3)	Paranoia (4)	Other (5)
Smoking (1)	☐	☐	☐	☐	☐
Vaping (2)	☐	☐	☐	☐	☐
Edibles (3)	☐	☐	☐	☐	☐
Tinctures (oil) (4)	☐	☐	☐	☐	☐
Topicals (5)	☐	☐	☐	☐	☐
Other (6)	☐	☐	☐	☐	☐

Page Break

5 Do you feel that cannabis with THC has consequences such as memory loss, loss of coordination, anxiety, and altered senses?

- ○ Yes (1)
- ○ No (2)

End of Block: THC Motives

Start of Block: Demographics

Q87 The following contains items pertaining to your demographic and background information. For each item, please respond with the appropriate information.

- ○ Proceed to questions (1)

Page Break

1 What is your biological sex?

- ○ Male (1)
- ○ Female (2)
- ○ Non-binary / third gender (3)
- ○ Prefer not to say (4)

---

2 How old are you in years?

____

---

3 Are you hispanic or latino?

- ○ Yes (1)
- ○ No (2)

---

4 What is your race?

- ○ American Indian or Alaska Native (1)
- ○ Asian (2)
- ○ Black or African American (3)
- ○ Native Hawaiian or Other Pacific Islander (4)
- ○ White (5)
- ○ Other (6)

---

5 How tall are you (in feet and inches; for example: 5'9")?

____

6 What is your weight (in lbs)?

____

7 What best describes your academic background?

- ○ High school diploma / GED (1)
- ○ Technical program certification (2)
- ○ Associates degree (3)
- ○ Undergraduate degree (4)
- ○ Graduate Degree Masters (5)
- ○ Graduate Degree Doctorate (6)
- ○ Other (7)

8 What best describes your yearly household income?

- ○ Less than $25,000 (1)
- ○ $25,000 - $99,999 (2)
- ○ $100,000 - $200,000 (3)
- ○ More than $200,000 (4)

9 In what country do you reside?

- ◯ United States and territories (1)
- ◯ Other (2) ____

Display This Question:

If In what country do you reside? = United States and territories

10 In which U.S. state or territory do you reside?

- ○ Alabama (1)
- ○ Alaska (2)
- ○ Arizona (3)
- ○ Arkansas (4)
- ○ California (5)
- ○ Colorado (6)
- ○ Connecticut (7)
- ○ Delaware (8)
- ○ District of Columbia (65)
- ○ Florida (9)
- ○ Georgia (10)
- ○ Hawaii (11)
- ○ Idaho (12)
- ○ Illinois (13)
- ○ Indiana (14)
- ○ Iowa (15)
- ○ Kansas (16)
- ○ Kentucky (17)
- ○ Louisiana (18)
- ○ Maine (19)
- ○ Maryland (20)

- Massachusetts (21)
- Michigan (22)
- Minnesota (23)
- Mississippi (49)
- Missouri (24)
- Montana (25)
- Nebraska (26)
- Nevada (27)
- New Hampshire (28)
- New Jersey (29)
- New Mexico (30)
- New York (31)
- North Carolina (32)
- North Dakota (33)
- Ohio (34)
- Oklahoma (35)
- Oregon (36)
- Pennsylvania (37)
- Rhode Island (38)
- South Carolina (39)
- South Dakota (40)

◯ Tennessee (41)

◯ Texas (42)

◯ Utah (43)

◯ Vermont (50)

◯ Virginia (44)

◯ Washington (45)

◯ West Virginia (46)

◯ Wisconsin (47)

◯ Wyoming (48)

◯ American Samoa (51)

◯ Baker Island (52)

◯ Guam (53)

◯ Howland Island (54)

◯ Jarvis Island (55)

◯ Johnston Atoll (56)

◯ Kingman Reef (57)

◯ Midway Atoll (58)

◯ Navassa Island (59)

◯ Northern Mariana Islands (60)

◯ Palmyra Atoll (61)

◯ Puerto Rico (62)

- United States Virgin Islands (63)
- Wake Island (64)

End of Block: Demographics
